# Surgical management of mediastinal liposarcoma extending from hypopharynx to carina: Case report

**DOI:** 10.1186/1477-7819-8-13

**Published:** 2010-03-02

**Authors:** Thomas L Gethin-Jones, Nathaniel R Evans, Christopher R Morse

**Affiliations:** 1 Division of Thoracic Surgery, Massachusetts General Hospital, Blake 1570, 55 Fruit St, Boston, MA 02114, USA

## Abstract

We describe the complete resection of a giant, well-differentiated mediastinal liposarcoma extending retropharynx to envelop the aortic arch, trachea and esophagus following preoperative radiotherapy.

## Background

Liposarcomas represent only 1% of all malignancies and are commonly found in the lower limbs and retroperitoneum [1]. Rarely are liposarcomas found in the mediastinum and, of all primary mediastinal sarcomas only 9% are liposarcomas [2]. Several reports suggest radiation and chemotherapy without surgical resection are ineffective treatments for mediastinal liposarcoma despite often daunting preoperative imaging [1,3]. In this case we report on the surgical resection of a large primary mediastinal liposarcoma by sternotomy.

## Case presentation

**Figure 1:**
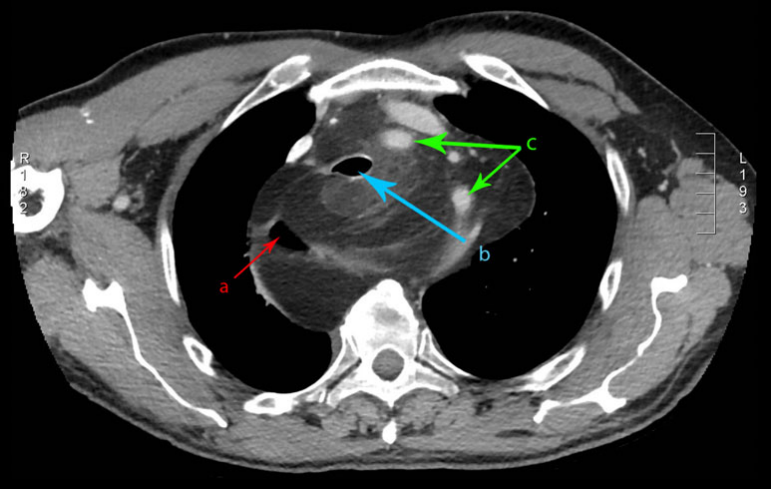
**Axial CT image of the mediastinal liposarcoma**. (a) indicates the position of the esophagus, (b) indicates the trachea, and (c) demonstrates the arch vessels.

**Figure 2:**
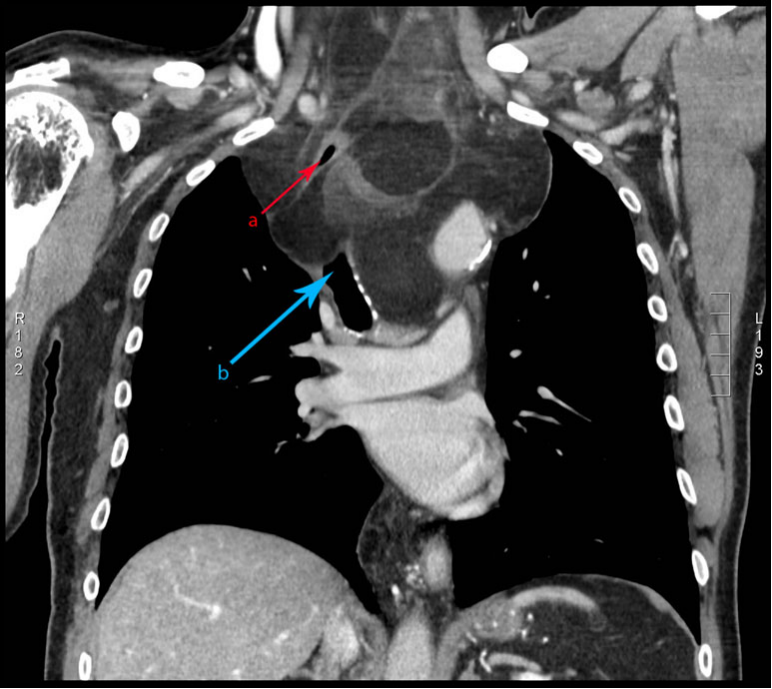
**Coronal CT images of well-differentiated mediastinal liposarcoma**. (a) indicates the position of the esophagus and (b) indicates the position of the trachea.

A 70-year-old male with no history of radiotherapy presented with gradual swelling of the neck and dyspnea of 7 to 8 months duration. Magnetic resonance imaging (MRI) and computed tomography (CT) scans of the neck and chest revealed a large mass extending from the hypopharynx to the carina (Figures 1 &2), causing significant displacement of the larynx, trachea, and esophagus as well as encasing the aortic arch. Fine needle aspiration (FNA) biopsy returned well-differentiated liposarcoma. Improvement of symptoms came with 10 cycles of neoadjuvant radiotherapy prior to surgical resection.

**Figure 3:**
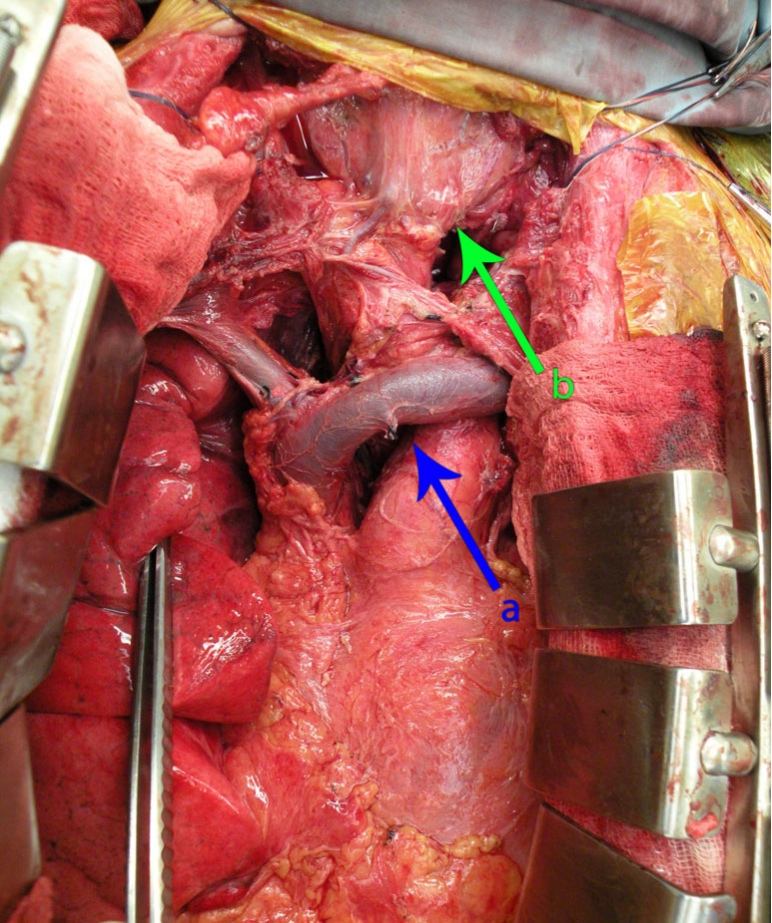
**Intraoperative photo following resection of well differentiated mediastinal liposarcoma**. (a) indicates the position of the innominate vein and (b) indicates the position of the trachea/larynx.

The patient was intubated while spontaneously ventilating and with rigid bronchoscopy available. Initial bronchoscopy revealed compression of the right mainstem bronchus. Passage of an upper gastrointestinal endoscope proved difficult with compression of the esophagus. Through an initial collar incision and with rotation of the carotid sheaths laterally, a well encapsulated 11 × 4 centimeter mass was dissected from behind the hypopharynx. As it extended far into the mediastinum, a sternotomy was performed and the left and right pleural spaces opened. The liposarcoma surrounded the aortic arch, and separated the trachea from esophagus. The tumor was dissected from under the brachiocephalic artery and rotated down from the neck. Laterally, a plane was identified along the esophagus and trachea, but the lesion was too large to move between the trachea and esophagus. Consequently, a lobulated portion of the mass was divided and removed through the right chest. A final component was dissected off the distal arch of the aorta to complete the resection (Figure 3). Postoperatively the patient was extubated and was discharged to home on postoperative day eight. He received postoperative radiation for a total of 60 Gy.

## Discussion

In the literature, less than 150 cases of primary mediastinal liposarcomas have been reported [1,4] and because of their rarity, there is no consistent approach to management. Warranting further study, radiology and chemotherapy alone seem to be insufficient forms of treatment but are possibly effective as induction or adjuvant therapies [1,2,5]. When determining if surgical intervention is feasible, radiographic films, given the complex anatomy of the mediastinum, can be daunting. However, given the often encapsulated nature of the lesions, complete resection is often possible and debulking can lead to symptomatic relief and often a long-term solution in well-differentiated tumors.

## Conclusions

Despite the complex nature of the anatomy surrounding mediastinal liposarcomas, surgical intervention is not unreasonable and thought to be the most effective form of treatment [1,3] especially in this particular case of an encapsulated, well-differentiated mediastinal liposarcoma.

## Competing interests

The authors declare that they have no competing interests.

## Authors' contributions

TLG-J helped draft the manuscript. CRM and NRE reviewed and edited the manuscript. All authors read and approved the final manuscript.

## Consent

Written informed consent was obtained from the patient for publication of this case report and any accompanying images. A copy of the written consent is available for review by the Editor-in-Chief of this journal.
